# Efficacy and safety of lasmiditan in patients using concomitant migraine preventive medications: findings from SAMURAI and SPARTAN, two randomized phase 3 trials

**DOI:** 10.1186/s10194-019-1032-x

**Published:** 2019-07-24

**Authors:** Li Shen Loo, Jessica Ailani, Jack Schim, Simin Baygani, Hans-Peter Hundemer, Martha Port, John H. Krege

**Affiliations:** 1 Lilly Research Laboratories, Lilly Corporate Center, Indianapolis, IN USA; 2 MedStar Georgetown University Hospital, Washington D.C., USA; 3 The Neurology Center of Southern California, Carlsbad, CA USA; 4 Lilly Deutschland GmbH, Medical Department, Bad Homburg, Germany

**Keywords:** Acute treatment, Concomitant, Ditan, Efficacy, Lasmiditan, Migraine, Migraine medication, Migraine preventive, Migraine prophylaxis

## Abstract

**Objective:**

To study the efficacy and safety of lasmiditan for acute treatment of migraine in patients using migraine preventive medications.

**Background:**

While lasmiditan has been proven to be an effective acute treatment for migraine, its effectiveness has not been examined when used concurrently with migraine preventives.

**Methods:**

SAMURAI and SPARTAN were similarly designed, double-blind, phase 3, placebo-controlled studies of patients 18 years or older with 3 to 8 migraine attacks per month. Patients were randomized to treat a migraine attack with oral lasmiditan 50 mg (SPARTAN only), 100 mg, 200 mg, or placebo. Migraine preventives were allowed as long as doses were stable for 3 months prior to screening and were unchanged during the study. Preventive medications with established or probable efficacy, as recommended by the American Academy of Neurology, the American Headache Society, and the European Headache Federation, plus botulinum toxin type A and candesartan, were included. Within the subgroups of patients using and not using preventive therapies, lasmiditan and placebo groups were analyzed for the outcome of pain-free at 2 h and other efficacy outcomes. The subgroups of patients using and not using preventive therapies were compared and interaction *p*-values were calculated for safety and efficacy outcomes.

**Results:**

In these trials, 698 of 3981 patients (17.5%) used migraine preventive treatments. Among patients using preventives, all lasmiditan doses resulted in significantly more patients being pain-free at 2 h, compared to placebo (*p* < 0.05). Primary efficacy outcome (pain-free at 2 h), key secondary outcome (most bothersome symptom-free at 2 h) and all other efficacy outcomes were not significantly different between patients using or not using migraine preventives (all interaction *p*-values ≥0.1). Rates of adverse events were similar for patients using and not using preventive medications.

**Conclusions:**

Lasmiditan was more effective than placebo for the acute treatment of migraine in patients concurrently using migraine preventive medications. Lasmiditan efficacy and safety measures were similar for patients using and not using preventive medications.

**Trial registration:**

SAMURAI (NCT02439320) and SPARTAN (NCT02605174). Registered 18 March 2015.

**Electronic supplementary material:**

The online version of this article (10.1186/s10194-019-1032-x) contains supplementary material, which is available to authorized users.

## Background

Migraine is the leading cause of disability in people under 50 years of age [1] and the second highest cause of disability worldwide with significant impact on the daily lives of patients and their families [2]. Migraine disease management includes preventive treatments to reduce attack frequency and acute medications to treat attacks. The American Migraine Prevalence and Prevention (AMPP) study found that an estimated 39% of patients with migraine experience attack frequency or migraine-related disability that would make them eligible for preventive treatments [3, 4]. Multiple classes of drugs are currently used to prevent migraine attacks including antiepileptic, antidepressant, and beta-adrenoceptor blocking medications [5]. The American Academy of Neurology (AAN) and the American Headache Society (AHS) developed evidence-based recommendations to help guide the use of migraine preventive medications in clinical practice [4], as has the European Headache Federation (EHF) [6]. These groups found that anti-epileptic medications, topiramate and valproic acid, as well as the beta-blockers, propranolol and metoprolol, have the highest levels of evidence supporting their efficacy. The anti-depressants, amitriptyline and venlafaxine, as well as some additional beta-blockers, were rated lower, but were still considered to be efficacious as migraine preventives.

Preventive treatments do not typically eliminate all migraine attacks. Patients using preventive drugs also need to use acute medication, making the concomitant use of preventive and acute migraine medications common. The most commonly recommended acute treatments for migraine include triptans and non-steroidal anti-inflammatory drugs (NSAIDs) [6]. However, not all patients respond sufficiently to triptans and NSAIDs, and in some patients, triptans are contraindicated due to their vasoconstrictive effects [7]. Lasmiditan is a novel serotonin receptor agonist, distinct from triptans in that it is selective for the 5-hydroxytryptamine (5HT)_1F_ receptor and does not result in vasoconstriction [8].

Lasmiditan has been proven to be effective in the acute treatment of migraine [9, 10], but the effectiveness of lasmiditan has not been examined when used concurrently with migraine preventive medications. The efficacy and safety of lasmiditan were studied in two similarly designed phase 3 clinical trials, SAMURAI [9] and SPARTAN [10], and included in these studies were patients on stable doses of concomitant migraine preventive medication. The objective of the post hoc analysis presented in this report was to investigate the efficacy and safety of lasmiditan for the acute treatment of migraine in patients using concomitant migraine preventive medications, compared with patients not using migraine preventives.

## Methods

### Phase 3 clinical trials

This study was a post hoc analysis of the pooled data from two randomized, double-blind, placebo-controlled phase 3 studies, SAMURAI [9] and SPARTAN [10]. The primary objective of each trial was to evaluate the efficacy of lasmiditan versus placebo in treating migraine-related headache pain and most-bothersome symptom (MBS). An in-depth description of study design and clinical results has been reported for SAMURAI [9] and SPARTAN [10].

Study protocols were approved by an independent ethics committee or institutional review board at each research site and both studies were conducted in accordance with the International Conference on Harmonization Good Clinical Practice guidelines as well as local regulatory requirements and conformed to the principles of the Declaration of Helsinki. Written informed consent was obtained from all patients before participation in the studies. Both studies were registered at ClinicalTrials.gov (SAMURAI identifier: NCT02439320, SPARTAN identifier: NCT02605174). The SAMURAI trial took place in the United States and SPARTAN involved locations in the United States, the United Kingdom, and Germany.

Trial participants were male or female, 18 years or older, with a diagnosis of episodic migraine with or without aura, fulfilling the International Classification of Headache Disorders-II diagnostic criteria 1.1 or 1.2.1 [11]. All enrolled patients had experienced migraine for at least 1 year and had a Migraine Disability Assessment (MIDAS) total score ≥ 11. Patients reported 3 to 8 migraine attacks monthly. Patients using migraine preventive medication were allowed to enroll if they had been on stable doses for the 3 months prior to screening and did not alter their treatment during the study.

Study designs were similar for both trials. Patients were evenly distributed into three treatment groups for SAMURAI: lasmiditan 100 mg, lasmiditan 200 mg, and placebo. In SPARTAN, a fourth group was added, lasmiditan 50 mg. Patients were asked to treat their next migraine attack with study drug within 4 h of pain onset.

Using an electronic diary, each patient entered assessments of their migraine attack at baseline and at 0.5, 1, 1.5, 2, 3, 4, 24, and 48 h after treatment with study drug. At these time intervals, patients recorded their level of headache pain as none, mild, moderate, or severe, and the presence or absence of migraine-associated symptoms, including nausea, vomiting, phonophobia, and photophobia. Patients identified their baseline MBS from choices of nausea, phonophobia, or photophobia. For each time point, patients also recorded their level of migraine-associated disability by responding to the question “How much is your migraine interfering with your normal activities?”, and response options were “not at all,” “mild interference,” “marked interference,” or “need complete bed rest.” At 2 h post-dose, patients also reported their global impression of change (PGIC) from baseline by answering the question “How do you feel after taking study medication?” using one of 7 response options: very much better, much better, a little better, no change, a little worse, much worse, or very much worse. The diary also queried how the patient was feeling daily and during attacks, and answers suggestive of possible adverse events were followed up with telephone calls from the sites to determine if adverse events had occurred.

The safety population included all randomized patients who took a dose of study medication. The intent-to-treat (ITT) population included all patients in the safety group who recorded a post-dose evaluation of their migraine-related symptoms, and the modified ITT (mITT) population included all patients from the ITT group who took study drug within 4 h of the onset of migraine headache pain. As was pre-specified, all analyses in this report were conducted using the ITT population, except for pain freedom and MBS freedom, which were conducted using the mITT population.

The primary efficacy outcome of the trials was the proportion of patients who reported having no headache pain (pain-free) at 2 h and the key secondary endpoint was the absence of MBS (MBS-free) at 2 h, after taking the study treatment.

Other secondary efficacy outcomes included pain relief (reduction in headache pain from “moderate” or “severe” at baseline to “mild” or “none” or from “mild” at baseline to “none”) at 2 h, sustained pain freedom at 24 h (absence of headache pain at 2 h that was sustained at 24 h without using a second dose of study drug or another migraine medication), total migraine freedom (absence of migraine headache pain and other migraine symptoms including nausea, phonophobia, photophobia, and vomiting) at 2 h, disability-free (migraine-related disability rated as “none”) at 2 h, and a PGIC rating of “very much better” or “much better” at 2 h.

### Post hoc analysis

#### Migraine preventive use

At the screening visit, patients reported their use of medications during the past 3 months as well as a complete history of migraine medication use. A list of migraine preventive medications was made, which included medications recommended by AAN, AHS, and EHF for the prevention of migraine [4–6]. Botulinum toxin type A and candesartan were also included because of support for their efficacy in recent studies [12–16]. Triptans were excluded due to their common use as an acute treatment for migraine. The complete list included

- divalproex sodium, sodium valproate, topiramate (anti-epileptics)
- metoprolol, propranolol, timolol, atenolol, nadolol (beta-blockers)
- amitriptyline, venlafaxine (anti-depressants)
- botulinum toxin type A, and
- candesartan.

Patients were considered to be “using migraine preventive medication” if they were taking any of the listed medications, regardless of their reason for use.

Subgroup analyses compared the group of patients using migraine preventive medications with the group not using migraine preventive medications. For efficacy outcomes, comparisons between subgroups were performed for individual lasmiditan dosing groups (50, 100, 200 mg) as well as placebo, whereas for safety outcomes, all lasmiditan groups were combined. Logistic regression modeling was used with factors including study, subgroup, treatment, and subgroup-by-treatment interaction. The interaction terms were examined for evidence of differential treatment effect dependent on subgroup level; interaction *p*-values less than 0.1 were considered significant. Additional comparisons were performed within the subgroups of those using and not using migraine preventive medications to examine the efficacy of lasmiditan doses versus placebo in the population of patients using migraine preventive medications. Mantel-Haenszel odds ratios, 95% confidence intervals, and general association *p*-values at each measured time point, stratified by study, were calculated for each subgroup category.

Two of the most commonly used migraine preventive medications, topiramate and propranolol, were pre-specified in the trials for subgroup analysis. In addition to analyses of subgroups of patients based on the full list of preventives, the following subgroups were analyzed for the efficacy outcomes of pain-free and MBS-free at 2 h post-dose.

#### Topiramate/propranolol use

Patients using topiramate and/or propranolol were compared with patients using no preventive or any preventive other than topiramate or propranolol.

#### Topiramate use

Patients using topiramate were compared with patients not using topiramate.

#### Propranolol use

Patients using propranolol were compared with patients not using propranolol.

Data were analyzed using the SAS 9.4 software (SAS Institute Inc., Cary, NC, USA).

## Results

### Patient demographics

A total of 3,981 patients were enrolled in the two trials, received a dose of study treatment, and recorded a post-dose evaluation of their migraine symptoms (ITT population). Of these patients, 17.5% were using migraine preventive treatments. The demographic characteristics of patients using and not using migraine preventive medications are shown in Table 1. On average, patients using preventive medications were 4 years older, had been living with migraine for 3 years longer, and were more likely to be female and white than patients who were not using migraine preventives (*p* < 0.01).

**Table 1:** Demographic characteristics of patient groups using and not using migraine preventive treatments

Patient characteristic (ITT population)	Using preventive treatments *N* = 698	Not using preventive treatments *N* = 3283
Age, mean (SD), years	45.7 (11.6)^**‡**^	41.4 (12.5)
Female, n (%)	613 (87.8)^**†**^	2742 (83.5)
Race, white, n (%)	595 (85.2)^**‡**^	2560 (78.0)
BMI, mean (SD)	30.3 (7.3)	30.2 (8.8)
Duration of migraine history, mean (SD), years	21.4 (13.8)^**‡**^	18.1 (12.6)
Average migraine attacks/month in past 3 months, mean (SD)	5.3 (1.8)	5.2 (1.9)

*BMI* body-mass index, *ITT* intent to treat, *N* number of patients in subgroup of ITT population, *n* number of patients with stated characteristic, *SD* standard deviation

^†^*p* < 0.05; ^‡^*p* < 0.001 compared with not using preventives

Of the 698 participants using one or more preventive treatments, 576 (82.5%) were using a single preventive and 122 (17.5%) were using 2 or more preventives. Medications in the anti-epileptic, beta-blocker, and antidepressant drug classes accounted for 93% of all migraine preventive treatments used by trial participants. Anti-epileptic use was most prevalent, making up 35% of all preventives used, followed by beta-blockers (32.3%), and antidepressants (25.5%). Botulinum toxin type A accounted for 5.7% of preventives used and candesartan for 1.4%.

Some migraine preventive medications can also be used to treat other disease conditions. Of 3,981 patients in the ITT population, 424 patients (10.7%) were recorded as using one or more of the listed preventives strictly for a migraine indication, while 213 patients (5.4%) were reported as using one or more of the listed preventives strictly for non-migraine indications. Another 61 patients (1.5%) were using one or more preventives for both migraine and non-migraine indications.

### Headache characteristics

A comparison of baseline characteristics of treated migraine attacks (Table 2) revealed that the two groups had some differences in baseline migraine severity. Compared with patients not using preventives, patients using preventives were less likely to report severe migraine attack at baseline and were more likely to rate their baseline severity as moderate (*p* < 0.05). The two groups had similar distributions for migraine-related disability at the time of treatment, though patients using preventives were somewhat more likely than patients not using preventives to report mild interference (*p* < 0.05). The percentages of patients experiencing nausea, phonophobia, photophobia, and vomiting at the time of attack were similar for patients using and not using preventive medications, as was the time that elapsed from the onset of migraine headache pain to the time of treatment.

**Table 2:** Baseline characteristics of treated migraine attacks for patients using and not using migraine preventive medications

Characteristic (ITT population)	Using preventive treatments *N* = 698	Not using preventive treatments *N* = 3283
Baseline migraine severity		
Severe, n (%)	174 (24.9)^†^	973 (29.6)
Moderate, n (%)	511 (73.2)^†^	2257 (68.7)
Mild, n (%)	13 (1.9)	52 (1.6)
Time to dosing from onset of pain in hours, mean (SD)	1.9 (4.3)	1.9 (4.3)
Baseline symptoms		
Photophobia, n (%)	537 (76.9)	2522 (76.8)
Phonophobia, n (%)	439 (62.9)	2048 (62.4)
Nausea, n (%)	316 (45.3)	1416 (43.1)
Vomiting, n (%)	14 (2.0)	80 (2.4%)
Baseline disability score		
Need complete bedrest, n (%)	118 (16.9)	585 (17.8)
Marked interference, n (%)	363 (52.0)	1795 (54.7)
Mild interference, n (%)	209 (29.9)^†^	862 (26.3)
Not at all, n (%)	8 (1.1)	41 (1.2)

*ITT* intent-to-treat, *N* number of patients in subgroup of ITT population, *n* number of patients with stated characteristic, *SD* standard deviation

^†^*p* < 0.05 compared with not using preventives

### Primary and key secondary efficacy outcomes

**Fig. 1:**
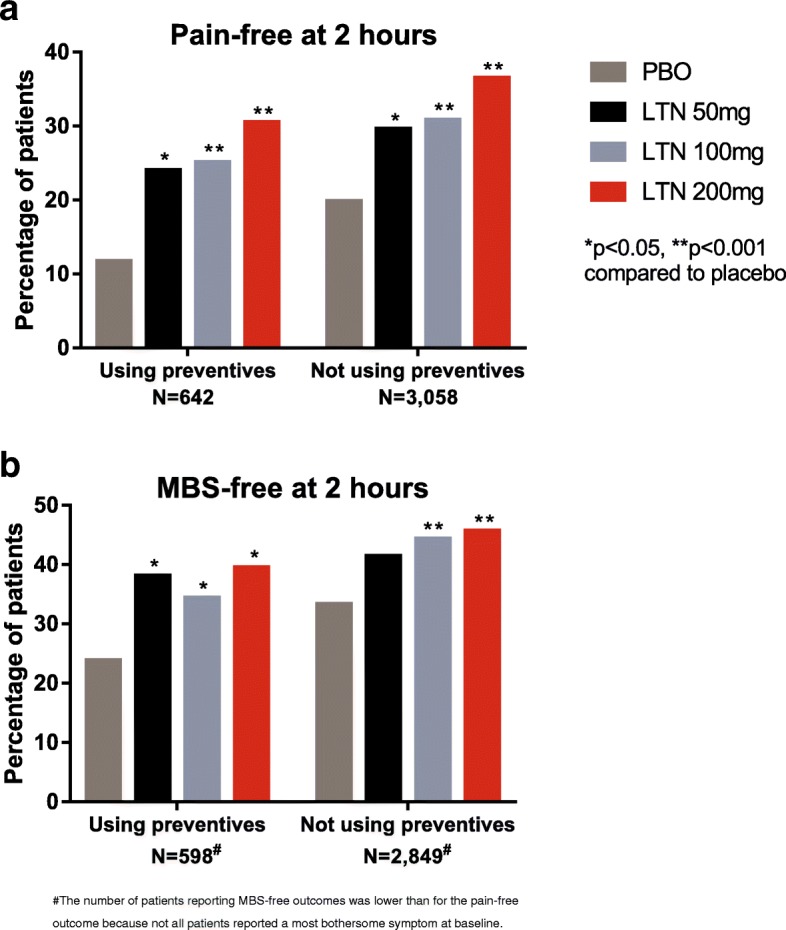
Pain-free and most bothersome symptom-free (MBS-free) at 2 h following lasmiditan treatment were similar in patients using and not using migraine preventive medications. Patients using and not using migraine preventive medications treated a migraine headache with lasmiditan (LTN) 50 mg, 100 mg, or 200 mg or placebo (PBO). At 2 h postdose, patients rated their pain and presence or absence of nausea, phonophobia, or photophobia. Colored bars show the percentages of patients reporting a complete absence of pain (**1a**) or MBS (**1b**) at 2 h. Comparisons of lasmiditan effect in the group of patients using versus not using preventive medications were not significant for any treatment group for either pain-free or MBS-free (all interaction *p*-values > 0.1)

Pain-free and MBS-free at 2 h were the primary and key secondary outcomes used to evaluate the efficacy of lasmiditan. The percentages of patients achieving pain freedom and MBS freedom are shown in Fig. 1. All doses of lasmiditan resulted in significantly greater (all *p*-values < 0.05) increases in the percentages of patients experiencing pain freedom and MBS freedom at 2 h, compared with placebo, both in patients using and not using preventives, with the exception of MBS freedom in patients not using preventives who treated with lasmiditan 50 mg (*p* = 0.08).

Although the percentages of patients meeting the primary outcomes in the group using preventive medications were generally lower than those observed in the group not using preventives, statistical comparisons between the two groups showed that the differences were not statistically significant for pain-free or MBS-free at 2 h (all interaction *p*-values > 0.1). The patients using preventives achieved pain-free and MBS-free outcomes at numerically lower percentages with placebo and lasmiditan treatment. However, the odds ratios comparing the percentages of patients achieving a response with lasmiditan versus placebo were similar or higher for the group using preventives than for the group not using preventives, for all doses of lasmiditan, for both pain-free and MBS-free at 2 h (Table 3).

**Table 3:** Odds ratios for lasmiditan treatment versus placebo were similar or greater in the group of patients using preventives than in those not using preventives

Outcome	Lasmiditan dose (mg)	Using preventives		Not using preventives		Interaction *p*-value^b^
		n/N (%)	Odds ratio^a^ (CI)	n/N (%)	Odds ratio^a^ (CI)	
Pain-free at 2 h	PBO	23/196 (11.7)		172/867 (19.8)		
	50	25/104 (24.0)	2.2 (1.0, 4.7)	134/452 (29.6)	1.4 (1.0, 1.9)	0.715
	100	44/175 (25.1)	2.5 (1.4, 4.4)	265/860 (30.8)	1.8 (1.4, 2.2)	0.654
	200	51/167 (30.5)	3.3 (1.9, 5.7)	321/879 (36.5)	2.3 (1.9, 2.9)	0.550
MBS-free at 2 h	PBO	45/189 (23.8)		271/813 (33.3)		
	50	37/97 (38.1)	2.0 (1.1, 3.7)	172/415 (41.4)	1.3 (1.0, 1.7)	0.342
	100	55/160 (34.4)	1.7 (1.0, 2.7)	358/809 (44.3)	1.6 (1.3, 1.9)	0.523
	200	60/152 (39.5)	2.1 (1.3, 3.3)	371/812 (45.7)	1.7 (1.4, 2.1)	0.674

*CI* confidence interval, *MBS* most bothersome symptom, *N* number of patients in the subgroup of mITT population, *n* number of patients achieving outcome, *PBO* placebo

^a^Odds ratio compared to patients who received placebo in the same subgroup

^b^Interaction *p*-value comparing patients using and patients not using migraine preventive medications

### Efficacy outcomes in patients using topiramate and propranolol

The two preventive medications pre-specified for subgroup analyses (topiramate and propranolol) were used by 340 (9%) of the 3,700 patients in the mITT population who treated a migraine attack. Pain-free and MBS-free outcomes at 2 h were similar for patients using topiramate/propranolol compared with patients not using these medications (interaction *p*-values > 0.1) (Additional file 1: Table S1). Subgroup analyses of data from patients using versus not using topiramate and of those from patients using versus not using propranolol also showed that results for pain-free and MBS-free at 2 h post-dose were similar for groups using and not using these preventives (data not shown).

### Additional secondary efficacy outcomes

Other efficacy outcomes, including pain relief, sustained pain freedom at 24 h, total migraine freedom, PGIC of “very much better” or “much better,” and disability-free at 2 h are shown in Table 4. Similar to the results for pain-free and MBS-free at 2 h, interaction *p*-values comparing patients using and not using migraine preventive medications were not significant (> 0.1) for all outcomes examined and for all doses of lasmiditan. Odds ratios for achieving a response among patients using preventives were similar to or greater than odds ratios among patients not using preventive treatments.

**Table 4:** Secondary efficacy outcomes were not significantly different between patients using and not using migraine preventive medications, for all doses of lasmiditan

Outcome	Lasmiditan dose (mg)	Using preventives		Not using preventives		Interaction *p*-value^b^
		n/N (%)	Odds ratio^a^ (CI)	n/N (%)	Odds ratio^a^ (CI)	
Pain relief, n/N (%)	PBO	88/207 (42.5)		420/922 (45.6)		
	50	61/113 (54.0)	1.5 (0.9, 2.5)	292/485 (60.2)	1.6 (1.3, 2.1)	0.630
	100	109/189 (57.7)	1.8 (1.2, 2.7)	595/944 (63.0)	2.0 (1.7, 2.5)	0.712
	200	114/189 (60.3)	2.1 (1.4, 3.1)	583/931 (62.6)	2.0 (1.7, 2.4)	0.567
Sustained pain freedom at 24 h, n/N (%)	PBO	11/196 (5.6)		99/867 (11.4)		
	50	12/104 (11.5)	1.7 (0.6, 4.4)	85/452 (18.8)	1.4 (1.0, 2.0)	0.660
	100	25/175 (14.3)	2.8 (1.3, 5.8)	150/860 (17.4)	1.6 (1.2, 2.2)	0.326
	200	30/167 (18.0)	3.7 (1.8, 7.7)	192/879 (21.8)	2.2 (1.7, 2.8)	0.293
Total migraine freedom, n/N (%)	PBO	18/196 (9.2)		159/867 (18.3)		
	50	23/104 (22.1)	3.1 (1.3, 7.4)	121/452 (26.8)	1.3 (1.0, 1.8)	0.437
	100	39/175 (22.3)	2.8 (1.6, 5.2)	241/860 (28.0)	1.7 (1.4, 2.2)	0.534
	200	43/167 (25.7)	3.4 (1.9, 6.2)	287/879 (32.7)	2.2 (1.7, 2.7)	0.604
PGIC very much better or much better, n/N (%)	PBO	39/207 (18.8)		243/923 (26.3)		
	50	36/113 (31.9)	2.0 (1.1, 3.8)	183/485 (37.7)	1.4 (1.1, 1.9)	0.830
	100	62/189 (32.8)	2.1 (1.3, 3.3)	382/944 (40.5)	1.9 (1.6, 2.3)	0.830
	200	69/189 (36.5)	2.5 (1.6, 3.9)	381/931 (40.9)	1.9 (1.6, 2.4)	0.421
Disability-free at 2 h, n/N (%)	PBO	34/201 (16.9)		222/912 (24.3)		
	50	29/113 (25.7)	1.6 (0.8, 3.2)	155/480 (32.3)	1.3 (1.0, 1.8)	0.751
	100	47/189 (24.9)	1.6 (1.0, 2.7)	320/934 (34.3)	1.6 (1.3, 2.0)	0.669
	200	54/187 (28.9)	2.0 (1.2, 3.2)	327/916 (35.7)	1.7 (1.4, 2.1)	0.631

*Abbreviations*: *CI* confidence interval, *MBS* most bothersome symptom, *N* number of patients in the subgroup of ITT population, *n* number of patients achieving outcome, *PBO* placebo, *PGIC* patient global impression of change

^a^Odds ratio compared to patients who received placebo in the same subgroup

^b^Interaction *p*-value comparing patients using and patients not using migraine preventive medications

### Efficacy of lasmiditan at time points up to 2 h

**Fig. 2:**
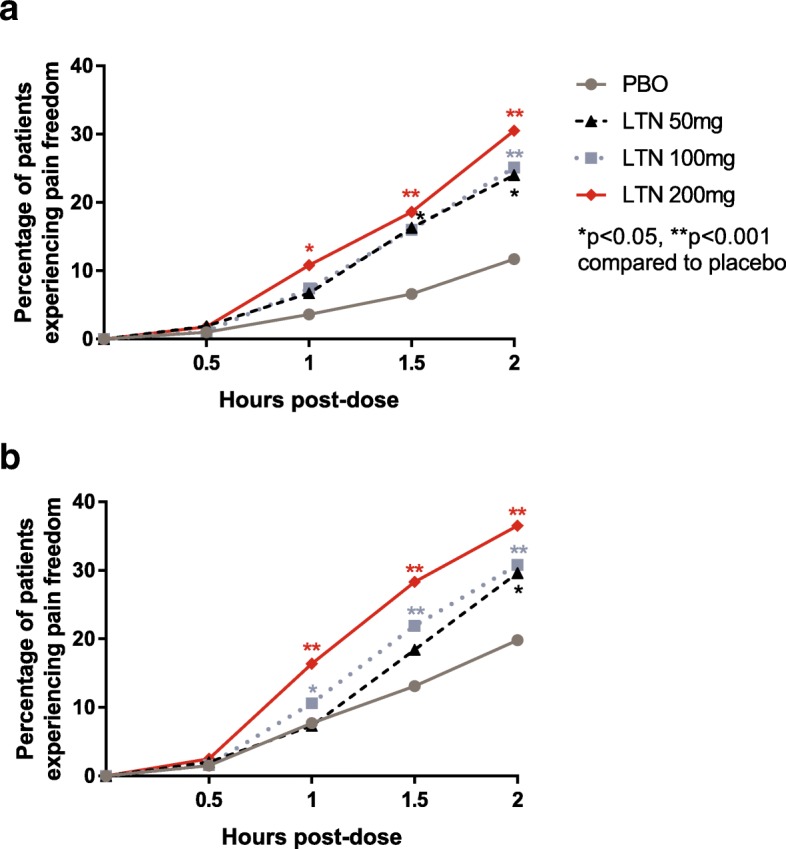
Pain freedom occurred at similar time points in patients using versus not using preventive medications. Patients using (**2a**) and not using (**2b**) migraine preventive medications treated a migraine attack with lasmiditan (LTN) 50 mg, 100 mg, or 200 mg of or placebo (PBO). They then rated their pain at 0.5, 1, 1.5, and 2 h postdose. The percentages of patients reporting no pain at each time point are shown in the graph. *p < 0.05 compared to PBO, **p < 0.001 compared with PBO

The percentages of patients achieving pain freedom at 0.5, 1, 1.5, and 2 h post-dose are shown in Fig. 2. In both groups of patients, lasmiditan treatment resulted in significantly more patients achieving pain freedom than with placebo, as early as 1 h after treatment for lasmiditan 200 mg (*p* < 0.05) and by 2 h for all doses of lasmiditan (*p* < 0.05). Overall, subgroup analysis revealed that the differences in percentages of patients achieving pain freedom between the groups of patients using and not using preventive medications were not statistically significant (all interaction *p*-values > 0.1) (Table 5).

**Table 5:** Pain freedom at 0.5, 1, 1.5, and 2 h did not differ significantly between groups using and not using migraine preventive medications

Hours after dosing	Lasmiditan dose (mg)	Using preventives		Not using preventives		Interaction *p*-value^b^
		Pain freedom n/N (%)	Odds ratio^a^ (CI)	Pain freedom n/N (%)	Odds ratio^a^ (CI)	
0.5	PBO	2/196 (1.0)		13/867 (1.5)		
	50	2/104 (1.9)	1.9 (0.2, 20.9)	9/452 (2.0)	1.3 (0.5,3.4)	0.714
	100	2/175 (1.1)	1.2 (0.2, 8.2)	14/860 (1.6)	1.1 (0.5,2.3)	0.904
	200	3/167 (1.8)	1.8 (0.3, 10.7)	22/879 (2.5)	1.7 (0.8, 3.4)	0.920
1.0	PBO	7/196 (3.6)		67/867 (7.7)		
	50	7/104 (6.7)	2.2 (0.6, 8.9)	33/452 (7.3)	0.9 (0.5, 1.5)	0.316
	100	13/175 (7.4)	2.2 (0.8, 5.6)	91/860 (10.6)	1.4 (1.0, 2.0)	0.854
	200	18/167 (10.8)	3.3 (1.3, 8.0)	144/879 (16.4)	2.3 (1.7, 3.2)	0.889
1.5	PBO	13/196 (6.6)		114/867 (13.1)		
	50	17/104 (16.3)	2.5 (1.0, 6.3)	83/452 (18.4)	1.3 (0.9, 1.8)	0.196
	100	28/175 (16.0)	2.7 (1.3, 5.3)	188/860 (21.9)	1.8 (1.4, 2.4)	0.732
	200	31/167 (18.6)	3.2 (1.6, 6.4)	249/879 (28.3)	2.6 (2.0, 3.3)	0.656
2.0	PBO	23/196 (11.7)		172/867 (19.8)		
	50	25/104 (24.0)	2.2 (1.0, 4.7)	134/452 (29.6)	1.4 (1.0, 1.9)	0.715
	100	44/175 (25.1)	2.5 (1.4, 4.4)	265/860 (30.8)	1.8 (1.4, 2.2)	0.654
	200	51/167 (30.5)	3.3 (1.9, 5.7)	321/879 (36.5)	2.3 (1.9, 2.9)	0.550

*Abbreviations CI* confidence interval, *N* number of patients in the subgroup of mITT population, *n*, number of patients achieving outcome, *PBO* placebo

^a^Odds ratio compared to patients who received placebo in the same subgroup

^b^Comparing subgroups of patients who were using and not using migraine preventive medications for the stated time point and treatment group

### Safety

There were no notable differences in the rates or types of adverse events (AEs) in the group of lasmiditan-treated patients using preventives compared with those not using preventive treatments. There were no deaths in either group and percentages of patients experiencing AEs and serious adverse events (SAEs) were similar for both groups. Treatment-emergent adverse events (TEAEs) also did not differ significantly between patients using and not using migraine preventive medications. The percentages of patients experiencing any of the most frequent TEAEs are shown in Table 6. Patients using preventives experienced SAEs at rates of 0% with placebo and 0.2% with lasmiditan treatment, whereas patients not using preventives experienced SAEs at rates of 0.3% with placebo and 0.3% with lasmiditan treatment.

**Table 6:** Treatment-emergent adverse events occurred at similar rates in patients using and not using migraine preventive treatments

TEAE	Lasmiditan dose (mg)	Using preventives	Not using preventives
		n/N (%)	n/N (%)
Dizziness	PBO	6/231 (2.6)	31/1031 (3.0)
	All LTN	81/544 (14.9)	385/2633 (14.6)
Paresthesia	PBO	2/231 (0.9)	17/1031 (1.6)
	All LTN	44/544 (8.1)	136/2633 (5.2)
Somnolence	PBO	5/231 (2.2)	22/1031 (2.1)
	All LTN	22/544 (4.0)	153/2633 (5.8)
Fatigue	PBO	2/231 (0.9)	6/1031 (0.6)
	All LTN	16/544 (2.9)	104/2633 (3.9)
Nausea	PBO	3/231 (1.3)	17/1031 (1.6)
	All LTN	21/544 (3.9)	86/2633 (3.3)
Muscular Weakness	PBO	0/231 (0.0)	0/1031 (0.0)
	All LTN	3/544 (0.6)	39/2633 (1.5)
Hypoesthesia	PBO	1/231 (0.4)	2/1031 (0.2)
	All LTN	3/544 (0.6)	36/2633 (1.4)

*N* number of patients in the subgroup of safety population, *n* number of patients with TEAE, *TEAE* treatment-emergent adverse event, All LTN, pooled population receiving lasmiditan (LTN) 50 mg, 100 mg, or 200 mg LTN

Treatment-by-subgroup interaction did not indicate any statistically significant interaction for any TEAE (all interaction *p*-values > 0.1)

## Discussion

Lasmiditan is a new type of medication for acute treatment of migraine with a mechanism and safety profile distinct from other migraine medications, including the commonly used triptans [8]. Previous studies have examined co-administration of an acute medication of the triptan class with a single preventive treatment [17–20]. The current analysis is the first to investigate the efficacy and tolerability of lasmiditan in patients using a range of concomitant migraine preventive medications with established or probable efficacy. Of the patients using migraine preventives, more than 17% were using 2 or more preventive treatments during the trial. Some patients were using botulinum toxin type A, a treatment generally reserved for patients with chronic migraine; these patients may have met the trial requirement of 3 to 8 migraine attacks per month because of their use of botulinum toxin type A. This post hoc analysis is important because patients using versus not using preventives may have differential responses to acute treatment. The results show that the efficacy and safety of lasmiditan in patients using and not using migraine preventives were similar.

Patients using preventives had an overall lower response to placebo compared with those not using preventives. Differences in patient demographics and disease history may have contributed to the lower placebo effect in the patients using migraine preventives. Patients using preventive medications were, on average, about 4 years older and had a 3-year longer duration of migraine history compared with those who did not use preventives. As patients using preventive medications had more experience with migraine treatments, they may have been less susceptible to placebo effect. However, interaction *p*-values indicate that the responses to lasmiditan were statistically similar in patients using and not using preventive treatments. Furthermore, the odds ratios were similar in both groups or higher in patients using preventives compared with patients not using preventives.

Other acute medications for migraine have also been shown to be effective when used concomitantly with preventive treatments. One study showed that acute treatment with triptans was equally effective in patients using and not using topiramate for migraine prevention [17]. Other studies demonstrated the efficacy of rizatriptan and sumatriptan as acute treatments for migraine in patients also taking a single preventive medication [18, 19].

We also analyzed the primary and key secondary outcomes in the group of patients using propranolol and/or topiramate (Additional file 2: Table S2), in patients using propranolol (data not shown), and in patients using topiramate (data not shown). Similar to the results of our main analysis comparing patients using versus not using established preventives, there were no significant differences between groups of patients using and not using these specific preventives, for pain-free and MBS-free efficacy outcomes.

A drug-drug interaction study of lasmiditan with topiramate (NCT03308669) [21] was conducted to assess the safety, tolerability, and pharmacokinetics of lasmiditan when coadministered with topiramate. Another drug-drug interaction study was conducted to investigate the effect of lasmiditan on heart rate and blood pressure in subjects receiving propranolol (NCT03270644) [22]. The results from these studies will be reported separately.

In identifying the population of patients considered to be using migraine preventive medications, we included medications recommended by the AAN, AHS, and EHF as having sufficient evidence supporting their efficacy [4–6]. We also included botulinum toxin type A because it is supported by several clinical trials [16, 23, 24] and was included in recent guideline updates by AAN and EHF [14, 25]. Furthermore, we included candesartan, which has been demonstrated to be effective as a migraine preventive in two independent, placebo-controlled studies [12, 13].

In the AMPP study, 12.4% of people with migraine used a migraine preventive medication, and another 17.2% of patients reported using medications with potential antimigraine effects but taken for other medical purposes [26]. In the pooled analysis of SAMURAI and SPARTAN, 17.5% of patients reported using a medication that met the study definition of a migraine preventive. This proportion of patients is similar to the 15–19% of patients using migraine preventive medication in a study examining the efficacy of sumatriptan [27].

A limitation of this post hoc analysis is that there were too few patients using each specific treatment to perform subgroup analyses for each individual preventive agent. However, the consistent effect of lasmiditan in the subgroup of patients taking preventives, and in the relatively large subgroups taking topiramate, propranolol, or either topiramate or propranolol, suggests general efficacy and tolerability across a variety of preventive agents. Another limitation is that lasmiditan 50 mg was included in only one of the two trials used in this analysis.

## Conclusions

As patients are frequently treated with both acute and preventive migraine medications, it is clinically relevant to assess whether the effects of acute medications for migraine differ in patients taking migraine preventives. The results of this analysis indicate that lasmiditan was effective and had no increased safety or tolerability issues in patients using concomitant migraine preventive medications available during the trials.

## Additional file

Additional file 1: **Table S1.** Odds ratios for lasmiditan treatment versus placebo were similar or greater in the group of patients using topiramate and propranolol. (DOCX 14 kb)

Additional file 2: **Table S2.** Treatment-emergent adverse events occurred at similar rates in patients using and not using migraine preventive treatments for all doses of lasmiditan. (DOCX 16 kb)

## Data Availability

Lilly provides access to all individual participant data collected during the trial, after anonymization, with the exception of pharmacokinetic or genetic data. Data are available to request 6 months after the indication studied has been approved in the US and EU and after primary publication acceptance, whichever is later. No expiration date of data requests is currently set once data are made available. Access is provided after a proposal has been approved by an independent review committee identified for this purpose and after receipt of a signed data sharing agreement. Data and documents, including the study protocol, statistical analysis plan, clinical study report, blank or annotated case report forms, will be provided in a secure data sharing environment for up to 2 years per proposal. For details on submitting a request, see the instructions provided at www.clinicalstudydatarequest.com.

## Abbreviations

AAN: American Academy of Neurology
AEs: Adverse events
AHS: American Headache Society
AMPP: American Migraine Prevalence and Prevention
EHF: European Headache Federation
ITT: Intent to treat
MBS: Most bothersome symptom
mITT: modified ITT
NSAIDs: Non-steroidal anti-inflammatory drugs
PGIC: Patient global impression of change
SAEs: Serious adverse events
TEAEs: Treatment-emergent adverse events
