# Supplementary material for: Efficacy and safety of lasmiditan in patients using concomitant migraine preventive medications: findings from SAMURAI and SPARTAN, two randomized phase 3 trials
Source: J Headache Pain. 2019 Jul 24;20(1):84. doi: 10.1186/s10194-019-1032-x (PMC6734212; doi:10.1186/s10194-019-1032-x)
Supplement: Supplementary file 1 — Table S1. Odds ratios for lasmiditan treatment versus placebo were similar or greater in the group of patients using topiramate and propranolol. (DOCX 14 kb) [file 10194_2019_1032_MOESM1_ESM.docx]

Additional file 1: Table S1. Odds ratios for lasmiditan treatment versus placebo were similar or greater in the group of patients using topiramate or propranolol

| Outcome | Lasmiditan dose (mg) | Using topiramate or propranolol | | Not using topiramate or propranolol | | Interaction p-value^†^ |
| --- | --- | --- | --- | --- | --- | --- |
|  |  | n/N (%) | Odds ratio^*^ (CI) | n/N (%) | Odds ratio^*^ (CI) |  |
| Pain-free at 2 h | PBO | 13/107 (12.1) |  | 182/956 (19.0) |  |  |
|  | 50 | 10/48 (20.8) | 1.8  (0.6, 5.4) | 149/508 (29.3) | 1.5  (1.1, 1.9) | 0.841 |
|  | 100 | 25/93 (26.9) | 2.6  (1.3, 5.5) | 284/942 (30.1) | 1.8  (1.5, 2.3) | 0.286 |
|  | 200 | 24/92 (26.1) | 2.6  (1.2, 5.4) | 348/954 (36.5) | 2.5  (2.0, 3.0) | 0.771 |
| MBS-free at 2 h | PBO | 24/103 (23.3) |  | 292/899 (32.5) |  |  |
|  | 50 | 17/45 (37.8) | 2.8  (1.1, 7.4) | 192/467 (41.1) | 1.3  (1.0, 1.7) | 0.381 |
|  | 100 | 28/83 (33.7) | 1.7  (0.9, 3.3) | 385/886 (43.5) | 1.6  (1.3, 1.9) | 0.861 |
|  | 200 | 29/86 (33.7) | 1.7  (0.9, 3.1) | 402/878 (45.8) | 1.8  (1.5, 2.1) | 0.577 |

CI, confidence interval; MBS, most bothersome symptom; N, number of patients in the subgroup of mITT population; n, number of patients achieving outcome; PBO, placebo

^*^Odds ratio compared to patients who received placebo in the same subgroup

^†^Interaction p-value comparing patients using and patients not using topiramate or propranolol.
