# Supplementary material for: Efficacy and safety of lasmiditan in patients using concomitant migraine preventive medications: findings from SAMURAI and SPARTAN, two randomized phase 3 trials
Source: J Headache Pain. 2019 Jul 24;20(1):84. doi: 10.1186/s10194-019-1032-x (PMC6734212; doi:10.1186/s10194-019-1032-x)
Supplement: Supplementary file 2 — Table S2. Treatment-emergent adverse events occurred at similar rates in patients using and not using migraine preventive treatments for all doses of lasmiditan. (DOCX 16 kb) [file 10194_2019_1032_MOESM2_ESM.docx]

Additional file 2: Table S2. Treatment-emergent adverse events occurred at similar rates in patients using and not using migraine preventive treatments for all doses of lasmiditan.

| TEAE | Lasmiditan dose (mg) | Using preventives | Not using preventives |
| --- | --- | --- | --- |
|  |  | n/N (%) | n/N (%) |
| Dizziness | PBO | 6/231 (2.6) | 31/1031 (3.0) |
|  | 50 | 11/123 (8.9) | 45/531 (8.5) |
|  | 100 | 28/205 (13.7) | 166/1060 (15.7) |
|  | 200 | 42/216 (19.4) | 174/1042 (16.7) |
|  | All LTN* | 81/544 (14.9) | 385/2633 (14.6) |
| Paresthesia | PBO | 2/231 (0.9) | 17/1031 (1.6) |
|  | 50 | 4/123 (3.3) | 12/531 (2.3) |
|  | 100 | 17/205 (8.3) | 56/1060 (5.3) |
|  | 200 | 23/216 (10.6) | 68/1042 (6.5) |
|  | All LTN* | 44/544 (8.1) | 136/2633 (5.2) |
| Somnolence | PBO | 5/231 (2.2) | 22/1031 (2.1) |
|  | 50 | 2/123 (1.6) | 33/531 (6.2) |
|  | 100 | 13/205 (6.3) | 52/1060 (4.9) |
|  | 200 | 7/216 (3.2) | 68/1042 (6.5) |
|  | All LTN* | 22/544 (4.0) | 153/2633 (5.8) |
| Fatigue | PBO | 2/231 (0.9) | 6/1031 (0.6) |
|  | 50 | 5/123 (4.1) | 13/531 (2.4) |
|  | 100 | 7/205 (3.4) | 45/1060 (4.2) |
|  | 200 | 4/216 (1.9) | 46/1042 (4.4) |
|  | All LTN* | 16/544 (2.9) | 104/2633 (3.9) |
| Nausea | PBO | 3/231 (1.3) | 17/1031 (1.6) |
|  | 50 | 7/123 (5.7) | 11/531 (2.1) |
|  | 100 | 7/205 (3.4) | 33/1060 (3.1) |
|  | 200 | 7/216 (3.2) | 42/1042 (4.0) |
|  | All LTN* | 21/544 (3.9) | 86/2633 (3.3) |
| Muscular Weakness | PBO | 0/231 (0.0) | 0/1031 (0.0) |
|  | 50 | 1/123 (0.8) | 6/531 (1.1) |
|  | 100 | 0/205 (0.0) | 16/1060 (1.5) |
|  | 200 | 2/216 (0.9) | 17/1042 (1.6) |
|  | All LTN* | 3/544 (0.6) | 39/2633 (1.5) |
| Hypoesthesia | PBO | 1/231 (0.4) | 2/1031 (0.2) |
|  | 50 | 0/123 (0.0) | 2/531 (0.4) |
|  | 100 | 0/205 (0.0) | 17/1060 (1.6) |
|  | 200 | 3/216 (1.4) | 17/1042 (1.6) |
|  | All LTN* | 3/544 (0.6) | 36/2633 (1.4) |

N, number of patients in the subgroup of safety population; n, number of patients with TEAE; TEAE, treatment-emergent adverse event; All LTN, pooled population receiving lasmiditan (LTN) 50 mg,100 mg, or 200 mg

*Treatment-by-subgroup interaction did not indicate any statistically significant interaction for any TEAE (all interaction p-values >0.1).
